# Supplementary material for: Acute high folic acid treatment in SH-SY5Y cells with and without MTHFR function leads to gene expression changes in epigenetic modifying enzymes, changes in epigenetic marks, and changes in dendritic spine densities
Source: PLoS One. 2021 Jan 7;16(1):e0245005. doi: 10.1371/journal.pone.0245005 (PMC7790414; doi:10.1371/journal.pone.0245005)
Supplement: Attachment — Submitted filename: Response to Reviewers.docx [file pone.0245005.s002.docx]

Response to Reviewers 10-12-2020

We thank the reviewers for taking the time to perform a rigorous review of our work during what is a busy and trying time for everyone! We think we have addressed the concerns brought forth in the review as follows:

Addressing editorial comments:

1) We have updated the formatting of our manuscript document to reflect the formatting requests of the journal.
2) We have formatted images to be better quality, and the images now meet the requirements for figures set forth by the journal.

Addressing reviewer comments:

1) We agree that evaluating the effects of rescuing the *MTHFR* knockdown would be an interesting investigation. Unfortunately, we do not have the funding/resources to do these experiments at the moment, but we have addressed the reviewer’s comment by suggesting this as a future study in the discussion and conclusions section.
2) We agree that evaluating DNA hydroxymethylation and expression of TET enzymes would benefit this paper, and we did have the resources readily available to carry out such experiments. Most of the changes in our manuscript are due to adding (1) a study on DNA hydroxymethylation levels (global 5-hmC), (2) a study on the gene expression levels of *TET1, TET2,* and *TET3* using Taqman assays, and (3) a study on enzyme activity for TETs.
3) We agree that evaluating the effects of the knockdown with and without the excess folic acid on a stem cell population would be an extremely interesting study. Unfortunately, repeating our experiment in a stem cell population was not an option at the moment due to financial constraints. We did address the reviewer comment by suggesting a study on stem cell differentiation, survival, and epigenetic marks as a potential future direction in our discussion and conclusions section.
4) We reformatted our images to be higher quality images with properties that align with the journal’s instructions.

Again, we thank the reviewers for their time.

Sincerely,

Kimberly R. Shorter, Ph.D. (corresponding author)
